# Survey data on orientations, boot camps, and pre-matriculation programs in schools/colleges of pharmacy

**DOI:** 10.1016/j.dib.2021.106938

**Published:** 2021-03-03

**Authors:** Eytan A. Klausner, Karen S. Mark, Erica L. Rowe, Beverly S. Hamilton

**Affiliations:** a Department of Pharmaceutical Sciences, South College School of Pharmacy, 400 Goodys Lane, Knoxville, TN 37922, United States of America; b Department of Math and Science, Lincoln Memorial University, 6965 Cumberland Gap Parkway, Harrogate, TN 37752, United States of America

**Keywords:** Orientation, Boot camp, Pre-matriculation program, Pharmacy education, Healthcare education

## Abstract

A survey about orientations, boot camps, and pre-matriculation programs in schools/colleges of pharmacy was approved by the South College Institutional Review Board (IRB). The survey was sent electronically to Assistant/Associate Deans of Academic Affairs or administrators in similar positions at schools/colleges of pharmacy in October 2016. The survey was closed two months later, in December, with 50 responses. The data that was collected from the survey included characteristics and components of orientations, boot camps, and pre-matriculation programs, such as session content and the frequency sessions appeared. The survey also collected descriptive information from respondents regarding certain demographics related to their schools/colleges of pharmacy (e.g., public or private institutions, a 4-year program or a 3-year program). The data can be used by schools/colleges of pharmacy and other healthcare professions that wish to revise or establish orientations, boot camps, and pre-matriculation programs.

## Specifications Table

**Table:** 

Subject	Pharmaceutical Sciences
Specific subject area	Pharmacy
Type of data	Primary data, Tables
How data were acquired	A survey was sent electronically to Assistant/Associate Deans of Academic Affairs or administrators in similar positions in schools/colleges of pharmacy using Qualtrics. The survey appears in Supplementary material 1.
Data format	Raw, Cleaned and Analyzed
Parameters for data collection	A survey was sent to 138 Assistant/Associate Deans of Academic Affairs or administrators in similar positions in schools/colleges of pharmacy.
Description of data collection	A Qualtrics survey was sent to email addresses of school administrators. Participants were advised that submission of the survey will be interpreted as their informed consent to participate in this project.
Data source location	South College School of Pharmacy, Knoxville, Tennessee, United States of America (USA)
Data accessibility	Data are included in this article

## Value of the Data

• The data shows characteristics of orientations, boot camps, and pre-matriculation programs of schools/colleges of pharmacy in the USA. In addition, the data describes various components and the frequency that each component appears in such programs. The data can be used as a platform for the construction of new, or modify established orientations, boot camps, and pre-matriculation programs.
• The data can benefit schools/colleges of pharmacy and other healthcare professions that wish to evaluate and/or revise existing orientations, boot camps, and pre-matriculation programs.
• The data may inspire further research into characterization and refinement of orientations, boot camps, and pre-matriculation programs. Such programs are considered beneficial for students of various healthcare professions [1], [2], [3], [4]. Enhancements of orientations, boot camps, and pre-matriculation programs may further support diverse students from various backgrounds who matriculate into programs of healthcare professions.

## 1 Data Description

The data are survey results obtained from administrators in 50 schools/colleges of pharmacy in the USA [5], see Supplementary material 2. Supplementary material 1 shows the survey that was sent to schools/colleges of pharmacy. Table 1 shows basic information regarding schools/colleges of pharmacy whose administrators participated in the study (*n* = 50). This information includes certain demographics such as public versus private ownership, length of program (traditional, 4-year program versus accelerated, 3-year program), accreditation status, and year of program establishment. Table 2 presents the characteristics of orientations, boot camps, or pre-matriculation programs. This descriptive information includes which type of program is offered, if any, and the lengths and attendance policies of such programs. Table 3 comprises a diverse list of sessions that appear in orientations, boot camps, or pre-matriculation programs and the frequency in which they appear. The various sessions are grouped into four major session domains: (1) time management organization, critical thinking & problem solving; (2) professionalism and ethics; (3) personal interactions; and (4) curriculum and scientific review content.

**Table 1:** Basic information regarding schools/colleges of pharmacy whose administrators participated in the study (*n* = 50).

Survey questions	% responses
Is your school/college a public or a private institution?	
Public	54
Private	46
Is your school/college a 4-year program or a 3-year program?	
4-year program	82
3-year program	10
Other	8
What is the accreditation status of your school/college?	
Fully accredited	98
Candidate status	2
Pre-candidate status	0
What year was the school/college of pharmacy founded?	
2015-present	0
2010–2014	12
2005–2009	16
2000–2004	8
Prior to 2000	64

**Table 2:** Characteristics of orientations, boot camps, or pre-matriculation programs.

Survey questions	% responses
Please indicate if your school/college has an orientation, a boot camp, or a pre-matriculation program (select all that apply) (*n* = 50)	
Orientation	98
Boot camp	10
Pre-matriculation program	12
Considering to administer in the next few years	12
How long is your orientation program? (*n* = 48)	
< 3 days	50
3–5 days	46
> 1 week	4
How long is your boot camp or pre-matriculation program? (Select all that apply) (*n* = 11)	
< 3 days	18
3–5 days	46
> 1 week, but < 2 weeks	0
2 weeks or longer	27
Occurs later in the first quarter/semester	9
What is the expectation of student attendance at the boot camp or pre-matriculation program? (*n* = 35)	
Mandatory	80
Expected	11
Left to students	9

**Table 3:** Sessions that appear in orientations, boot camps, or pre-matriculation programs and their frequency (*n* = 48).

Sessions included (select all that apply)	% responses
Time management organization, critical thinking & problem solving	
Time management & organizational skills	71
Test taking & study skills	60
Critical thinking & problem solving skills	35
Concept of co-curriculum & associated activities	48
Introduction to drug information resources	15
Accessing, using, and citing resources	15
Professionalism and ethics	
Professionalism	85
Review of academic standards and integrity	79
Professional communication skills	44
Identifying and avoiding plagiarism	54
Dress code/fashion show	54
Introduction to professional organizations	77
Cultural diversity & awareness	46
Personal interactions	
Importance of leadership	50
Team work & team building activities	69
Interactions with upperclassmen student pharmacists	65
Mentor (faculty) - mentee (student) interactions	58
Interactions with faculty	73
Interactions with alumni	31
Curriculum and scientific review content	
Curriculum overview	88
Experiential education overview	79
Chemistry	10
Biochemistry	6
Anatomy and physiology	12
Microbiology	4
Math/calculations	8
Statistics	4
Medical terminology	8

## 2 Experimental Design, Materials and Methods

A survey instrument aimed to support a separate project of the authors regarding a pre-matriculation program [6] was designed by the authors. The goal of the survey was to collect data to identify similarities and differences concerning orientations, boot camps, and pre-matriculation programs in schools/colleges of pharmacy in the USA. The survey covered three main topics: (1) the characteristics of orientations, boot camps, and pre-matriculation programs; (2) the sessions that appear in such programs and how frequent they appear; and (3) certain demographics of schools/colleges of pharmacy whose administrators responded to the survey. Email addresses of Assistant/Associate Deans of Academic Affairs or administrators in similar positions in 138 schools/colleges of pharmacy were obtained from their respective websites. Qualtrics was used to send the survey to email addresses of administrators in October 2016. The survey was closed in December 2016. One reminder was sent to potential participants. While the data were collected anonymously, a survey question gave the respondents an opportunity to provide the name of their institution. Forty-eight from 50 respondents provided the name of their institution. Data were exported from Qualtrics to Microsoft Excel for processing and organization; as well as to produce a supplemental file and tables. No data were missing.

## Ethics Statement

The Institutional Review Board (IRB) of South College approved this study under the *Exempt* status. Participants in the study were communicated that submission of the survey will be interpreted as their informed consent to participate in this study.

## Declaration of Competing Interest

None declared.

## Supplementary materials

Image, application 1

Image, application 2

## Footnotes

Supplementary material associated with this article can be found in the online version at doi:10.1016/j.dib.2021.106938.
