# Supplementary material for: Survey data on orientations, boot camps, and pre-matriculation programs in schools/colleges of pharmacy
Source: Data Brief. 2021 Mar 3;35:106938. doi: 10.1016/j.dib.2021.106938 (PMC7967005; doi:10.1016/j.dib.2021.106938)
Supplement: Supplementary file 1 [file mmc1.docx]

Appendix 1. Survey about orientations, boot camps, and pre-matriculation programs in schools/colleges of pharmacy in the US

- What is the name of your school/college?

- Is your school/college a public or a private institution?

- Public

- Private

- Is your school/college a 4-year program or a 3-year program?

- 4 year program

- 3 year program

- Other

- What is the accreditation status of your school/college?

- Fully accredited

- Candidate

- Pre-candidate status

- What year was the school/college of pharmacy founded?

- 2015 – present

- 2010 – 2014

- 2005 – 2009

- 2000 – 2004

- Prior to 2000

- Please indicate if your school/college has an orientation, a boot camp, or a pre-matriculation program (select all that apply)

- Orientation

- Boot camp

- Pre-matriculation program

- School/college is considering the implementation of an orientation, boot camp, and/or a pre-matriculation program in the next few years

- N/A

- How long is your orientation program?

- Less than 3 days

- 3-5 days

- More than 1 week

- N/A

- How long is your boot camp or pre-matriculation program? (Select all that apply)

- Less than 3 days

- 3-5 days

- More than 1 week but less than 2 weeks

- 2 weeks or longer

- Occurs over the first quarter/semester of the first professional year

- N/A

- What is the expectation of student attendance at the boot camp or pre-matriculation program?

- Mandatory

- Expected

- Recommended but left for the student’s discretion

- From the following sessions, please indicate which are currently included in your orientation, boot camp, or pre-matriculation program? (Select all that apply.)

- Curriculum overview/graduation requirements

- Experiential education overview

- Scientific review of pre-requisite content

- Chemistry

- Biochemistry

- Anatomy and physiology

- Microbiology

- Math/calculations

- Statistics

- Medical terminology

- Time management and organizational skills

- Test taking and study skills

- Critical thinking and problem solving skills

- Introduction to drug information resources

- Accessing, using, and citing resources

- Identifying and avoiding plagiarism

- Professionalism (self-awareness, social media, etc.)

- Dress code/fashion show

- Review of academic standards and integrity

- Professional communication skills

- Importance of leadership

- Team work and team building activities

- Introduction to professional organizations

- Cultural diversity and awareness

- Concept of co-curriculum and associated activities

- Mentor (faculty) – mentee (student) interactions

- Interactions with upperclassmen student pharmacists

- Interactions with alumni

- Interactions with faculty

- Please comment about any other sessions in your program(s) that were not listed above
